# Overexpression of eIF3a in Squamous Cell Carcinoma of the Oral Cavity and Its Putative Relation to Chemotherapy Response

**DOI:** 10.1155/2012/901956

**Published:** 2012-04-23

**Authors:** Rita Spilka, Klaus Laimer, Felix Bachmann, Gilbert Spizzo, Alexander Vogetseder, Manuel Wieser, Heimo Müller, Johannes Haybaeck, Peter Obrist

**Affiliations:** ^1^Pathologie Labor Dr. Obrist & Dr. Brunnhuber OG, Klostergasse 1, 6511 Zams, Austria; ^2^Division of Maxillofacial Surgery, Innsbruck Medical University, 6020 Innsbruck, Austria; ^3^Basilea Pharmaceutica International Ltd., 4005 Basel, Switzerland; ^4^Department of Hematology and Oncology, Hospital Franz Tappeiner, 39012 Merano, Italy; ^5^Institute of Clinical Pathology, University Hospital Zürich, 8091 Zürich, Switzerland; ^6^Institute of Pathology, Medical University Graz, 8036 Graz, Austria

## Abstract

The eukaryotic translation initiation factor eIF3a is one of the core subunits of the translation initiation complex eIF3, responsible for ribosomal subunit joining and mRNA recruitment to the ribosome. It is known to play an important role in general translation initiation as well as in the specific translational regulation of various gene products, among which many influence tumour development, progression, and the therapeutically important pathways of DNA damage repair. Therefore, beyond its role in protein synthesis, eIF3a is emerging as regulator in tumour pathogenesis and therapy response and, therefore, a potential tumor marker. By means of a tissue microarray (TMA) for histopathological and statistical assessment, we here show eIF3a expression in 103 cases of squamous cell carcinoma of the oral cavity (OSCC), representing tissues from 103 independent patients. A subset of the study cohort was treated with platinum based therapy. Our results show that the 170 kDa protein is upregulated in OSCC and correlates with good overall survival. Overexpressing tumors respond better to platinum-based chemotherapy, suggesting eIF3a as a putative predictive as well as prognostic tumor marker in OSCC.

## 1. Introduction

Head and neck squamous cell carcinomas (SCCs) range at place six of the most common cancers worldwide. They are responsible for approximately 650,000 newly diagnosed tumors and an annual rate of 350,000 deaths [1]. Cancers of the oral cavity are a large subgroup (48%) of head and neck tumors, where more than 90% are SCCs [2]. The major risk factors for oral cancer development are smoking or chewing tobacco as well as alcohol abuse. In Asian countries, betel nut and gutkha quid consumption is responsible for exceptional high cancer prevalence [2]. Even if being independent risk factors, alcohol and tobacco show synergistic effects in increasing the risk of cancer development when used together. This is partially due to the fact that the consumption of alcohol increases the permeability of the oral mucosa which enables an enhanced effect of carcinogenic nitrosamines and polycyclic hydrogen contained in tobacco. Other risk factors are insufficient oral hygiene, chronic pressure by dental prostheses, and chronic diseases, including infection with human papilloma virus [3–5]. First diagnosis of oral squamous cell carcinomas (OSCC) is frequently at late disease stage, with two thirds of patients baring a tumor of stage III or IV. These tumors, therefore, go along with severe morbidity and a long-term survival lower than 50%, despite advances in surgery and conservative cancer treatments (radiotherapy, chemotherapy and immunotherapy) of oral cancer [6]. A need for new prognostic and predictive biomarkers is given and their investigation is regarded as essential to improve OSCC management. 

Current OSCC treatment is a single modality therapy for early stage and multimodality therapeutic strategy in cases of advanced tumors. A large number of early detected tumors will be subjected to surgical excision, as long-term sequelae associated with radiotherapy can be circumvented. Patients with late stage tumors usually have to undergo surgery in combination with preoperative or postoperative radiation therapy [7] Approaches with adjuvant chemotherapy are controversially discussed, with yet no common guidelines. Chemotherapy regimens consist of a combination of 5-fluorouracil (5-FU) with platinum-containing drugs, including cisplatin and carboplatin [8].

**eIF3a as Tumor Marker**

The eukaryotic translation initiation factor (eIF) 3a is a 170 kDa protein and presents the largest subunit of the eIF3 complex. eIF3 is involved in the translation initiation process at the formation of Met-tRNAi-40S preinitiation complex, and it regulates the recruitment of mRNA to this complex (via an interaction with eIF4G). eIF3 is responsible for the prevention of immature ribosomal subunit joining and scaffolds numerous translation initiation interactions in this rate-limiting step of protein synthesis [9]. eIF3a can be seen as key regulator bringing together mRNA and the ribosome. As the eIF3 complex is an assembly of up to 13 proteins, it still remains an open question which subunits determine the major functions of eIF3. eIF3a together with eIF3b, eIF3c, eIF3e, eIF3f, and eIF3h is known to form the functional core of the eIF3 multisubunit complex [10]. Opposing this fact, eIF3a was reported recently to be not essential for general translation, as its knockdown reduced protein synthesis for only 15–20% *in vitro* [11, 12]. In accordance with this finding, eIF3a was reported to be responsible for the specific translation of a set of proteins, of which p27, RRM2 and tyrosinated *α*-tubulin have already been determined [11, 13]. eIF3a is known to be upregulated in various cancer entities, ranging from breast, cervical, colon, esophageal to lung, and gastric cancers [14–19]. Of particular importance is the vast difference in results of the mentioned studies. Dellas et al. first described the overexpression of eIF3a to correlate with a better prognosis in SCC, represented by cervical neoplasias [18]. Another study conducted in SCC focused on esophageal cancer, again showing a correlation of high eIF3a expression with good clinical prognosis [16]. Similar to these studies, eIF3a expression was analysed in adenocarcinomas (ACs) with rather contrasting outcomes. In a previous study, we could show in colon cancer that high eIF3a expression correlates with poor prognosis [19]. This discrepancy between AC and SCC is expected to be originating from different patterns of dedifferentiation and recruitment of protein synthesis machinery in the different cancer types. Little is known about translational differences between AC and SCC up to now.We hypothezise that eIF3a and moreover its translational targets play a crucial role in the distinction between pathogenic protein synthesis regulation in SCC and AC. Currently, upregulation of eIF3a is believed to not involve the whole eIF3 complex suggesting a specific role of eIF3a in cancer development and progression ranging possibly beyond its prime protein synthetic function. It will be interesting to understand how eIF3a influences downstream proteins, probably by targeted translation, and thereby regulation of cell proliferation molecules, including the already identified cell cycle regulator p27, tyrosinated *α*-tubulin, and ribonucleotide reductase M2 subunit [11, 20, 21]. Recently, it was also reported that eIF3a modulates nucleotide excision repair (NER) protein functions [22, 23]. It was shown that upon knockdown of eIF3a, the expression of XPA, XPC, RPA32, and RAD23B is increased. eIF3a was reported to influence response to cisplatin therapy, which acts as a DNA-damaging agent via this pathway [22].

The aim of this study was to determine the differential expression of the translation initiation factor eIF3a in tumor tissues of OSCC. We hypothesized that upregulation of eIF3a correlates with carcinogenesis, therefore, representing a novel cancer biomarker. The study cohort enabled describing the effect of eIF3a upregulation in a subgroup receiving chemotherapy, which is important knowledge to confirm correlative data of eIF3a expression and consequences for NER, as have been published for other tumor entities [22, 23].

## 2. Materials and Methods

### 2.1. Patients

This retrospective study included 103 patients who were operated with OSCC at the Department of Maxillofacial Surgery, Innsbruck University Hospital in the years between 1980 and 1997. From all of them, clinical followup data are available. The tumor material was fixed in formalin and paraffin-embedded according to routine methods. The study was conducted in accordance to the regulations of the local ethics committee and Austrian law. The cohort includes 79 (76.7%) male and 24 (23.3%) female patients. Their range of age is 25–86 years, with a median of 63 years. Twenty-eight (27%) patients were treated by radiotherapy, 88.9% of these patients suffered from a high grade tumor. Twenty-eight (27%) patients received a platinum-based chemotherapy. A haematoxylin and eosin staining was performed on all slides of each clinical specimen and assessed independently by two experienced and board-certified pathologists (J. Haybaeck, P. Obrist) in a blinded manner, according to standard pathology criteria, for tumor grading and staging. A summary of pathological features is shown in Table 1.

### 2.2. TMA

The tissue microarray (TMA) paraffin blocks were manufactured by a maxillofacial surgeon (k. Laimer) and a pathologist (P. Obrist). Representative tumor areas were identified on a haematoxylin-eosin-stained tumor slide and in the following were punched out, at a diameter of 2 mm, of the original paraffin block. The collection of punched tumor cones were united in a blank recipient paraffin block employing a precision instrument for assembly (Manual Tissue Arrayer, MTA-1, Beecher Instruments, Sun Prairie, Wis.). In this study, 4 different TMA blocks were used, containing approximately 30 tumor cones, according to a specific pattern. The sections taken were 5 *μ*m thick, mounted on specific adhesive-coated glass slides, compatible for immunohistochemical staining and analysis.

### 2.3. Immunohistochemistry

The antibody used to determine expression of eIF3a was a monoclonal rabbit antibody (eIF3A (D51F4) XP Rabbit mAb number 3411, Cell Signaling Technology, Danvers, MA). Staining was essentially performed as previously described using the Dako Autostainer Universal Staining System [24]. Endogenous peroxide blocking was achieved with incubation of TMA slides in 3% hydrogen peroxide for 5 minutes. The primary anti-eIF3a antibody was applied at a dilution of 1 : 100, for 60 minutes, followed by incubation with peroxidise-labelled secondary antibody for 30 minutes and substrate-chromogen 3,3′-diaminobenzidine tetrahydrochloride for 8 minutes. Counterstaining was performed in aqueous haematoxylin for 45 seconds. 

eIF3a expression was evaluated in terms of signal intensity (intensity score) and density (proportion score). The intensity score describes the staining intensity in comparison to a control staining (0 no staining, 1 weak, 2 moderate, 3 high); proportion score represents the estimated fraction of positively stained tumor cells (0–100%).

### 2.4. Statistical Methods

All calculations and the statistical analyses were performed using the statistical software program SPSS for Windows (SPSS, Inc. Chicago, IL). Differences between groups were tested for statistical significance applying the *χ* ^2^ test. Kaplan-Meier statistics were used to describe and calculate survival curves. Patients who were lost during followup were censored in the followup time parameter. For this method, *P*-values were evaluated by the log-rank test for censored survival data. The significance of eIF3a status for chemotherapy sensitivity and difference in overall survival was additionaly calculated in a multivariate ANOVA. Interparameter correlations were assessed according to Spearman's nonparametric test. For all analyses, a *P*-value < 0.05 was defined as statistically significant.

## 3. Results

**Figure 1:**
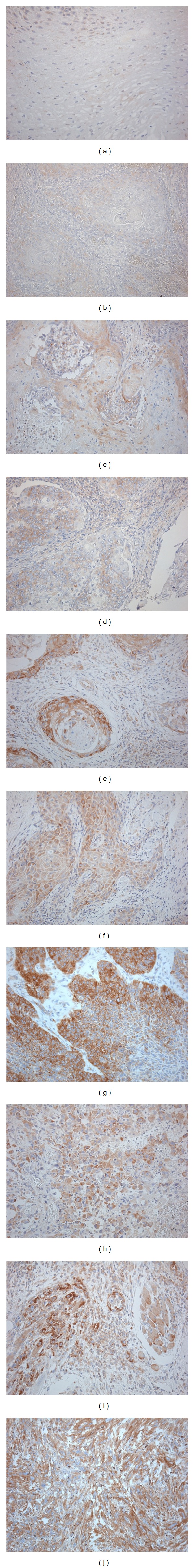
Microscopic pictures of the expression patterns of eIF3a in OSCC (200x). (a): Normal squamous epithelium with only slight inhomogeneous, mostly perinuclear eIF3a expression. (b): Low expression of eIF3a. (c/d) show a intermediate expression of eIF3a; (f–j) represent a high expression pattern of eIF3a.

The expression of eIF3a in the analysed OSCC TMA was evaluated by two pathologists (J. Haybaeck, P. Obrist). The intracellular eIF3a localisation was predominantly perinuclear, as can be expected to be the translationally active eIF3a in association to the endoplasmic reticulum. Even high-grade tumors were not completely positive for eIF3a. A zonal eIF3a expression distribution pattern was observed including regions with high, intermediate and low expression found adjacent to each other (Figure 1).

For statistical analyses, staining proportion score (PS) and intensity score (IS) were calculated reaching a total immunostaining score (TIS). To do so, PS values were categorised in the following way: no cells positively stained = 0, <20% = 1, 21–50% = 2, 51–80% = 3, >80% = 4. With these values, TIS was obtained by multiplication of IS and PS (TIS = IS × PS), resulting in TIS values ranging from 0 to 12, with only nine possible values (0, 1, 2, 3, 4, 6, 8, 9, 12). With this scoring system, statistics were calculated, based on the cut off that TIS ≥ 4 represents samples with an overexpression of eIF3a. In our cohort, 66% of samples showed an overexpression of eIF3a (Table 1). In detail, 2.8% had a score 0; 9.8% a score 1; 4.2% a score 2; 8.4% a score 3 and 4 each, the majority of 25.9% had an eIF3a score of 6 and 12.6% reached a score of 9.

**Figure 2:**
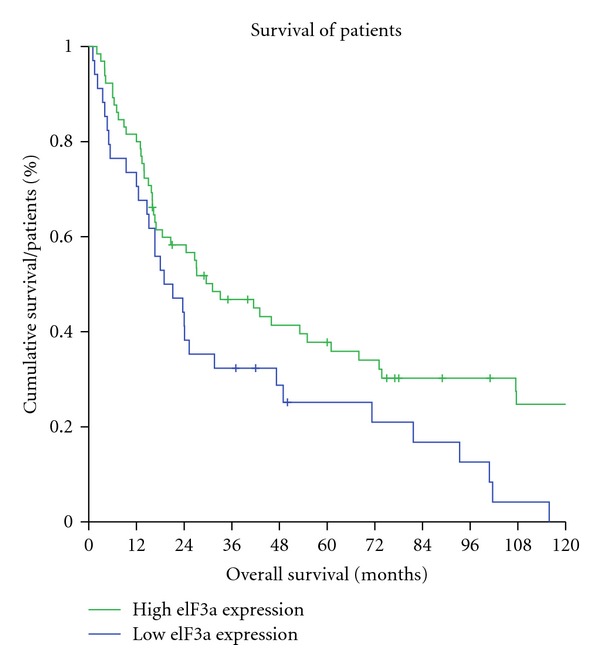
Kaplan-Meyer curve. Association of eIF3a expression with survival time in OSCC. The blue line indicates patients with eIF3a TIS > 4, the green line represents eIF3a overexpressing patients, with a TIS ≥ 4. The difference was significant in a log-rank test (*P* = 0.021).

The overall survival of patients in this study ranged from 1 to 245 months, with a median value of 27.2 months. The influence of eIF3a expression on overall survival was tested and blotted in a Kaplan Meyer curve (Figure 2) after having performed log-rank statistics. A clear difference is observed in patients with high and low eIF3a expression (*P* = 0.021), indicating that eIF3a overexpression is associated with a better overall survival. The average overall survival of patients baring eIF3a overexpressing tumors was 69 months (5.8 years), compared to 36 months (3 years) in patients with tumors of low eIF3a expression levels.

**Figure 3:**
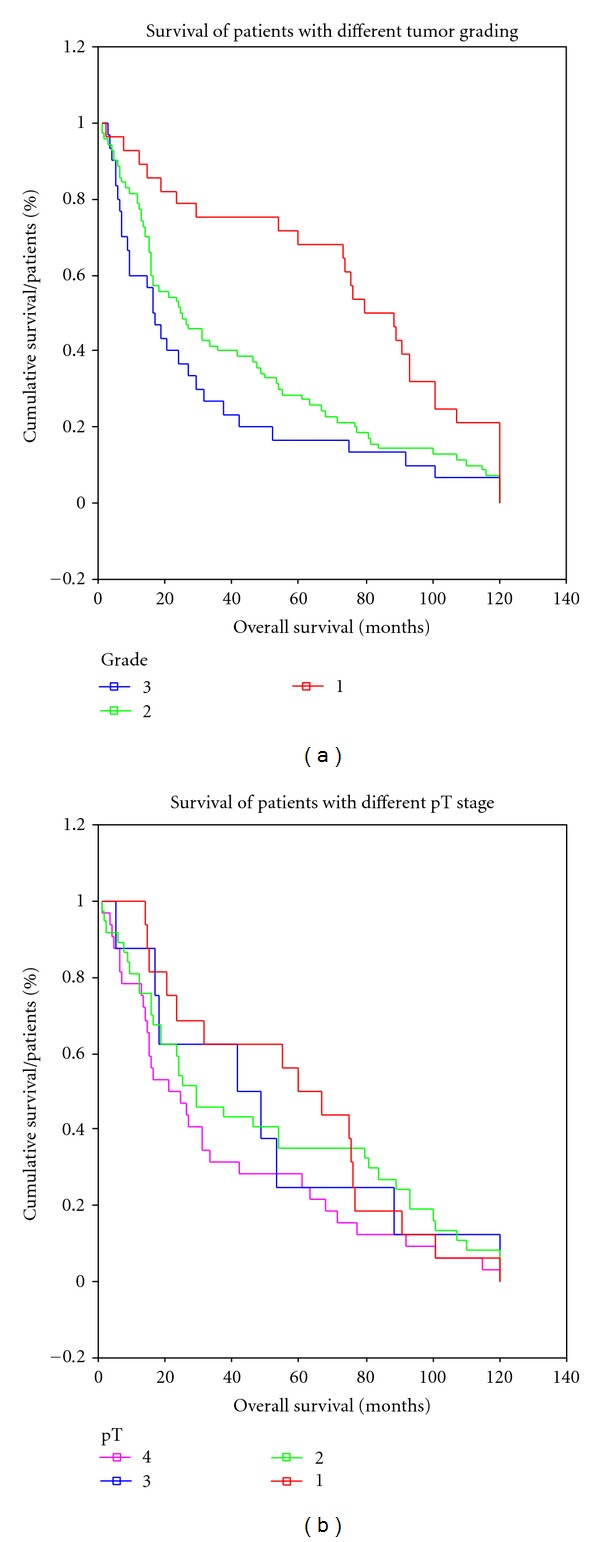
(a): Overall survival of patients with different tumor grading, (b): overall survival of patients with different tumor stage.

Our patient cohort is a representative set of OSCC patients, verified by a typical pattern of overall survival compared to tumor grade and stage (Figures 3(a) and 3(b)). 

**Figure 4:**
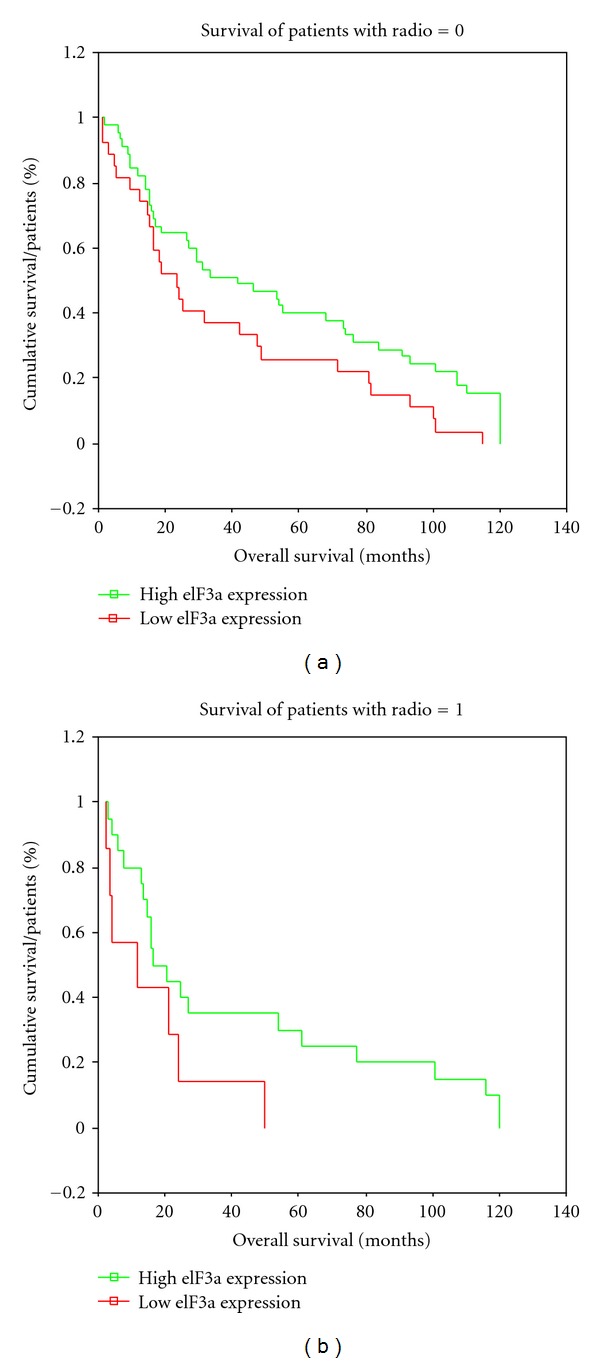
(a): Overall survival of patients with high and low eIF3a expression receiving no radiotherapy, (b): overall survival of patients with high and low eIF3a expression receiving radiotherapy.

Opposingly, we could not show any significant association of eIF3a expression with clinical stage (*P* = 0.948), tumor grade (*P* = 0.221), age (*P* = 0.452), sex (*P* = 0.055), and nodal status (*P* = 0.716). Expression of eIF3a also showed no significant difference in patients' response to radiotherapy (Figures 4(a) and 4(b)). The better overall survival of patients with high eIF3a expression is evident here, but not dependent on radiotherapy. As can be also seen in Figure 4, patients receiving no radiotherapy have a better overall survival, which is based on the fact that patients undergoing radiotherapy were mainly suffering from high grade tumors. This subgroup of patients receiving radiotherapy comprised to 11.1% G1, 58.3% G2, and 30.6% G3 tumors.

**Figure 5:**
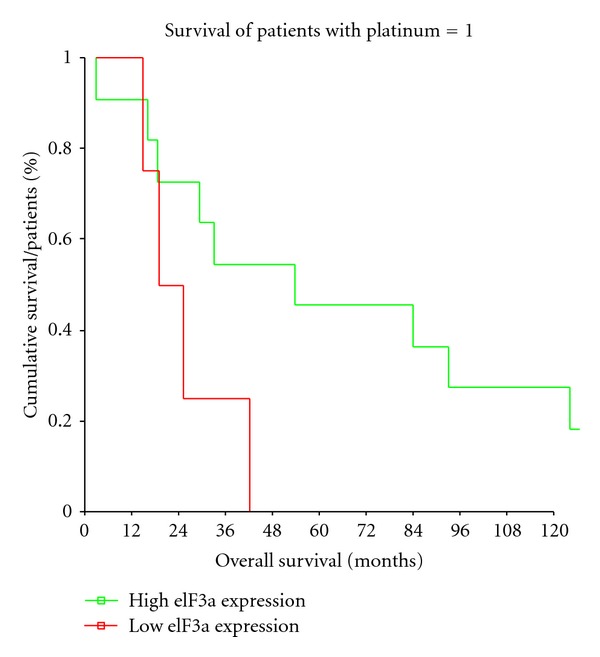
Kaplan-Meyer curve. Overall survival of patients receiving platinum based-chemotherapy. The red line represents patients with high eIF3a expression, the green line represents patients with low eIF3a expression (*P* = 0.037).

Further, a subgroup analysis was performed on patients receiving chemotherapy (*n* = 28). 72% of chemotherapeutically treated patients displayed an overexpression of eIF3a (TIS ≥ 4). The representative subgroup comprises 25% grade 1 (*n* = 7), 39% grade 2 (*n* = 11), and 36% grade 3 (*n* = 10) tumors, of 20 male (71%) and 8 female (29%) patients. The overall survival was analysed in those four groups and compared statistically by means of a multivariate ANOVA, revealing a significant difference in overall survival in patients receiving platinum-based chemotherapy, dependent on their eIF3a expression status (*P* = 0.034). The average overall survival for patients with high eIF3a levels was 67.2 and 53.3 years, with and without chemotherapy, respectively, and 27.9 and 35.7 years for patients with low eIF3a, with and without chemotherapy, respectively (Table 2; Figure 5).

## 4. Discussion

Squamous cell carcinoma of the oral cavity and oropharynx is a major representative of head and neck cancer, which accounts worldwide for approximately 4% of total carcinomas in men and 2% in women. These rates vary geographically. Prognosis of OSCC is based on TNM classified clinical staging and tumor grading [25]. In the past years, different strategies adding chemotherapy to radiotherapy were developed in order to improve treatment outcome [26]. Nevertheless, especially the identification of tumor markers as prognostic as well as predictive factors allows an accurate selection for assignation of patients to optimal therapy. 

In this study, we show that eIF3a is overexpressed in OSCC which correlates with a significant better prognosis of the patient. Analysing overall survival in patients receiving radiotherapy, we could not show a significant involvement of eIF3a expression status in therapy response rates. On the other hand, eIF3a overexpressing OSCC were identified to better respond to platinum-based chemotherapy.

The multisubunit translation initiation factor eIF3 is known to be orchestrated by 13 different proteins. All of these eIF3 proteins (eIF3a–eIF3m) are encoded on genetically different loci and were reported to assembly to different eIF3 subcomplexes [27]. 27 such subcomplexes are known to date and were characterized by mass spectrometry. eIF3a is part of 3 such subcomplexes, suggesting a putative independent mode of action [27]. This awareness to a special position of eIF3a among translation initiation factors initiated many studies investigating the role of eIF3a in a multitude of pathways, interactions, and cancer entities where it is intermingled. 

In a mouse model, eIF3a expression was analysed in various tissues and in the course of fetal and postnatal development [28]. It was shown that eIF3a is highly expressed in fetal compared to differentiated tissues. Strikingly, eIF3a expression, which was reduced in all analysed postnatal specimen, almost vanished in tissue samples of postnatal lung, intestine, and stomach [28]. Similar findings show that even in adult mice eIF3a expression in mucosal intestinal cells which are differentiated is low or absent whereas high expression patters are seen in basal intestinal cells of healthy adult mice [28]. *In vitro* assays of colon cancer cell lines indicated a similar dependence on decreased eIF3a expression for differentiation of cells [28]. Still, a thorough pathway and translational target analyses of how eIF3a is regulating early development and cellular differentiation is still lacking and should be expected with great interested from future studies.

This association of eIF3a with early development is paralleled with findings of an upregulation in cancer. *In vitro* studies, conducted in immortalized fibroblast cells, showed that the overexpression of eIF3a led to a malignant transformation of these cells [29]. This transformation manifested in the formation of transformed foci, suppression of apoptosis, and an increased in the measured polysomes to monosomes ratio [29]. These *in vitro* findings suggest that especially the pattern of specific translationally regulated proteins determines the transforming potential of immortalized fibroblasts and that eIF3a is recruiting a proteome capable of such malignant transformation. *In vivo*, eIF3a is found overexpressed in a range of tumor entities, where it was described in association with a rather contrastive clinical outcome. On the one hand, based on *in vitro* experiments in lung and breast cancer cell lines, eIF3a was suggested to be involved in the development of cancer and to be affiliated to sustained malignancy [13]. An association of eIF3a overexpression with poor clinical prognosis was reported recently in colon cancer patients following surgery [19]. This coherence and proclamation of eIF3a as procancer molecule is in line with the findings by Liu et al. on early mouse embryonic development and further based on the theory that eIF3a is regulating the translation of a specific, development- and differentiation-, as well as cancer-associated subset of mRNAs rather than global translation initiation. Known translational targets of eIF3a are the cell cycle regulators p27, tyrosinated *α*-tubulin, and ribonucleotide reductase M2 subunit, a key regulator of DNA synthesis [13]. On the other hand, studies in squamous cell carcinoma of the cervix and esophagus showed eIF3a expression to correlate with better prognosis. eIF3a expression was discussed to protect cells from progression into higher malignancy and to reduce metastatic potential of the respective tumors [16]. These findings underline that eIF3a is a key molecule of cancer development and progression, though not readily defined to date. Our finding that eIF3a upregulation interrelated to better overall survival in OSCC patients agrees with previous findings in other SCC. The diverse findings on eIF3a expression and associated clinical parameters are hypothesised to be highly dependent on tumor origin. Little is known about the differences of protein synthesis in AC versus SCC but our data corroborate a contrasting pattern and modality of translation (initiation) in which eIF3a seems to play a central role.

 The regulation of tyrosinated *α*-tubulin could link eIF3a expression to tumor therapy via the chemotherapeutic Docetaxel. Docetaxel is a tumor-therapeutic agent acting on the formation of microtubule and prevents physiological disassembly of microtubules, as required in mitotic cell division [11, 30]. A possible correlation of eIF3a expression and response to Docetaxel therapy should be analysed in followup studies. Preliminary, suggestive data is available, as it was recently shown the treatment of A549 lung cancer cells with docetaxel upregulates eIF3a expression *in vitro* [31]. Recently, a new group of eIF3a downstream targets that might have huge impact on tumor therapy decisions and introduce eIF3a as not only prognostic but also a predictive tumor marker that was discovered in the NER pathway. It is well known that tumor formation is often associated with impaired capability of DNA damage repair or cell cycle upon recognition of (spontaneous) mutations. Pathways required for these control mechanisms are drug-able in recent times, meaning that they may be targeted by chemotherapeutics. As example, platinum-based chemotherapeutics, like cisplatin or carboplatin, induce DNA double-strand breaks that need to be repaired by the complex process of NER. Main contributors to this pathway are xeroderma pigmentosum complementation group proteins (XPA, XPC), radiation-sensitive mutant 23 homolog B (RAD23B), as well as replication protein A (RPA). These were analysed for their eIF3a dependence in an *in vitro* model systems for nasopharyngeal carcinoma [22]. eIF3a was shown to negatively regulate the NER proteins and, therefore, to reduce DNA repair in cancer cells, which leads to increased apoptosis compared to cells with reduced eIF3a. Thus, elevated levels of eIF3a seem to hinder NER and thereby mutation incorporation and furthermore sensitizes cells to the treatment of DNA damaging agents [22]. These findings were verified in a patient cohort receiving platinum-based chemotherapy as first-line therapy against primary lung tumors, where tumor patients with high eIF3a expression responded significantly better to platinum-based chemotherapy [23]. In the present study, we found that eIF3a was highly expressed in responders in comparison to nonresponders of OSCC patients platinum-based chemotherapy. Here, it should be noted that, being assigned according to today's standard selection criteria, patients receiving cisplatin or carboplatin survived in average 53 months after diagnosis compared to 47 month in patients who did not undergo this treatment. This promotes the ongoing extensive investigation into discovery of novel biomarkers for suitability as molecular indicators of therapy response in OSCC patients. eIF3a was shown to be a potent biomarker fulfilling the stated requirement, as we found a significant correlation of eIF3a expression with better treatment response and increased overall survival. It is important to stress that in our findings, without eIF3a overexpression, a platinum-based therapy regimen would rather present a contraindication. Patients with low eIF3a expressing OSCC receiving platinum-based neo-djuvant therapy had a decreased overall survival of an average 8 months. Future fortification of these data is required by analysis of a bigger study cohort undergoing therapy. Platinum-based chemotherapeutics may be the first ones with a described dependence on a translational regulator as eIF3a. Nevertheless, the number of treatment strategies considering translation (initiation) as putative target is enormously increasing [32–34]. Rapamycin and novel analogue molecules, so-called Rapalogs, represent one class of putative tumor therapeutics which act by inhibiting protein synthesis via the mammalian target of rapamycin, mTOR. The kinase protein mTOR is activating protein synthesis by phosphorylation of downstream S6 kinase and 4E-binding proteins, which will in turn release eIF4E and trigger canonical translation initiation. Among eIFs, especially eIF4 and its subunits, represent interesting new targets for inhibition of translation initiation in tumor therapy [35–37]. eIF3 should not be neglected in this context, as the various compositions of the multisubunit complex, targeting different subsets of mRNAs for translation, represent target entities which might enable a more specific inhibition of tumor-related gene expression.

## 5. Conclusion

The investigation of predictive biomarkers helps differentiating likely responding patients from nonresponders. eIF3a was suggested to be a prognostic factor in OSCC with a high expression being associated with better clinical outcome. eIF3a might be a potent candidate as predictive tumor marker for evaluating sensitivity to platinum-based chemotherapy. To further validate eIF3a as a potential predictive and prognostic marker, future adjuvant trials using chemotherapy in addition to radiation therapy in oral squamous cell carcinoma should consider incorporating eIF3a in their correlative studies. Considering the many cancer entities where eIF3a is already described, this leaves eIF3a as a putative broad-range tumor marker which might still bear important keys for unlocking present issues in cancer diagnostics and therapy. 

## Figures and Tables

**Table 1:** Distribution of conventional clinico-pathological parameters of patients with OSCC in relation to eIF3a expression.

	eIF3a expression	
Parameter	Low (%)	high (%)

Number of patients	34	66
Sex		
Male	47	30
Female	19	4

Grading		
I	24.2	14.7
II	57.6	52.9
III	18.2	32.4

pN		
0	42.2	64.3
I	20.0	10.3
II	35.6	25.0
III	2.2	0

pT		
I	8	3
II	18	11
III	4	2
IV	17	10

**Table 2:** Overall survival in months of patients receiving platinum-based chemotherapy (1) or not (0), with respect to their eIF3a expression status.

	eIF3a expression	
	Low	High
Platinum-based chemotherapy	Average overall survival (months)	

1	67.2	27.9
0	53.3	35.7
